# Large‐Scale Synthesis of Monodispersed Perovskite Nanocrystals via Autonomous Continuous Droplet Microfluidics

**DOI:** 10.1002/advs.202524155

**Published:** 2026-01-31

**Authors:** Guangguang Huang, Xiangyu Liu, Long Song, Hailong Feng, Zuliang Du

**Affiliations:** ^1^ National & Local Joint Engineering Research Center for High‐efficiency Display and Lighting Technology Key Laboratory for Special Functional Materials of Ministry of Education School of Nanoscience and Materials Engineering Henan University Kaifeng China

**Keywords:** autonomous synthesis, monodispersity, Perovskite nanocrystals, Photoluminescence, zwitterionic ligands

## Abstract

The scalable synthesis of perovskite nanocrystals (PNCs) with monodispersity and high emission efficiency remains a significant challenge, primarily limited by the inherent constraints of flask‐based batch synthesis in handling large volumes. Here, we propose a novel microscale continuous droplet‐in‐flow synthesis (µ‐CDFS) of PNCs stabilized by zwitterionic ligands. This synergy, arising from the spatial and temporal separation of droplet‐based reactors and a cross‐locked PNC‐ligand surface coordination, ensures exceptional mixing efficiency and consistency. Consequently, our approach enables the scalable production of high‐quality PNCs, delivering a continuous output of 0.8 g h^−^^1^ per channel, a narrow size distribution (σ_r_ < 14%), and a photoluminescence quantum yield (PLQY) exceeding 97%. Furthermore, high‐throughput ligand screening is also performed via autonomous flow experimentation, and the emission of PNCs is precisely tuned across the visible region (453–753 nm) via on‐demand halide anion exchange, establishing a robust platform for the accelerated discovery of PNCs.

## Graphical abstract

In this work, we propose a novel on‐chip microscale continuous droplet‐in‐flow synthesis (μ‐CDFS) that utilizes zwitterionic ligands to enable large‐scale preparation of perovskite nanocrystals (PNCs) with high emission efficiency and monodispersity. This platform allows for on‐demand halide anion exchange and high‐throughput screening through sequential autonomous in‐flow experiments, thereby accelerating the discovery of PNCs.

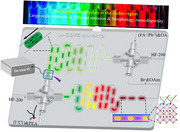

## 1 Introduction

Perovskite nanocrystals (PNCs), of the general formula APbX_3_ (A═Cs/FA; X═Cl/Br/I), have been studied intensively for applications in lighting and displays owing to their exceptional optoelectronic properties, such as high defect tolerance, narrow‐band emission, and large absorption coefficients [1, 2, 3]. Achieving size monodispersity in an ensemble of PNCs is the most critical factor for their basic scientific research and industrial applications [4, 5, 6]. However, compared to traditional II‐VI and III‐V covalent nanocrystals, the morphology control of ionic PNCs is relatively difficult due to their extraordinarily rapid reaction kinetics in several seconds, including organic‐inorganic hybrid FAPbX_3_ and all‐inorganic CsPbX_3_ [7, 8, 9]. After development for several years, the monodisperse PNCs have been well achieved via flask‐based batch synthesis (one‐pot hot‐injection and ligand‐assisted reprecipitation) [10, 11]. Nevertheless, this approach is typically limited to reaction volumes of several tens of milliliters. Scaling up the synthesis amplifies existing mixing inefficiencies, which adversely affect mass transfer kinetics and lead to size‐defocusing of the products. Additionally, the manual operation involved in flask‐based synthesis makes it challenging to ensure that all precursors follow consistent nucleation and growth pathways. Therefore, the scalable synthesis of monodisperse and highly efficient PNCs remains a significant challenge.

Microscale continuous droplet‐in‐flow synthesis (µ‐CDFS) has been demonstrated as an alternative strategy to the flask‐based synthesis [12, 13, 14]. Each droplet during µ‐CDFS serves as an independent reaction unit, eliminating back‐mixing of reaction solution while enabling precise process control. Thus, µ‐CDFS offers rapid mixing and high mass transfer rates, ensuring minimal batch‐to‐batch variation. Moreover, its scalable production is achieved by simply prolonging the reaction time, rather than by scaling up the reactor volume. Significant developments on u‐CDFS have been reported by Andrew J. deMello, Xiaobin Jiang, and Abolhasani et al., [15, 16, 17, 18]. For instance, Andrew J. DeMello's group unveiled the shape evolution and halide‐ion‐segregation in PNCs, and revealed the nature of molecular surface ligands used to synthesize and stabilize PNCs [15, 19]. Abolhasani's group systematically studied the flow synthesis of PNCs from rapid parameter space mapping to AI‐guided modular manufacturing, especially for emission regulation via ion exchange reactions [20, 21, 22]. Recently, Jiang's group proposed a multistage microfluidic‐based epitaxial growth strategy for type‐I core–shell PNCs [18]. Despite these advances in elucidating growth kinetics and optimizing ligands for strong emission, the application of µ‐CDFS for the large‐scale preparation of monodisperse PNCs remains largely unexplored.

Specifically, a successful large‐scale synthesis of PNCs via µ‐CDFS requires two prerequisites: (i) the consistent maintenance of PNC uniformity within each droplet all the time during flow, and (ii) the prevention of unfavorable Ostwald ripening after PNC growth. Unfortunately, the PNCs in crude solution are thermally unstable and typically necessitate terminating the reaction within several minutes via fast purification, which cannot meet the continuity of droplet microfluidics. Otherwise, the reaction progresses into the Ostwald ripening stage, resulting in a large size distribution, undesired phase transformation, or uncontrolled aggregations [23]. These factors pose significant obstacles to achieving monodisperse and efficient PNCs via µ‐CDFS.

To address this challenge, we developed a ligand‐assisted µ‐CDFS strategy for the large‐scale production of PNCs, which offered the following advantages: (i) effective suppression of Ostwald ripening via zwitterionic ligands (PEA: 1‐octyl‐2‐dodecanol‐3‐phosphoethanolamine; PPA: 1‐octyl‐2‐dodecanol‐3‐phosphopropanolamine), ensuring consistent product quality across droplets; (ii) enhanced mixing efficiency and mass transfer via autonomous droplet‐based microfluidics, enabling uniform nucleation and growth pathways for all precursors; and (iii) rapid parameter optimization through high‐throughput screening, accelerating the discovery and development of high‐performance PNCs. As a result, the green‐emitting PNCs (e.g., FAPbBr_3_ and CsPbBr_3_) were initially synthesized in flow with the PLQY >97%; then the color of PNCs can be switched “on demand” in the visible region (453–753 nm) via sequential anion exchange; a 7‐inch LCD based on R&G PNC@polymer backlights was fabricated with a wide color gamut reaching 130% of the National Television Systems Committee (NTSC) standard. Our µ‐CDFS demonstrated a high continuous PNC production capacity of 0.8 g h^−1^ per channel with a standard deviation of size distribution σ_r_ <14%, meeting the scale‐up of PNCs while maintaining efficient emission and monodispersity.

## 2 Results and Discussion

### 2.1 Computational Fluid Dynamics of Different Reactor Configurations

**FIGURE 1:**
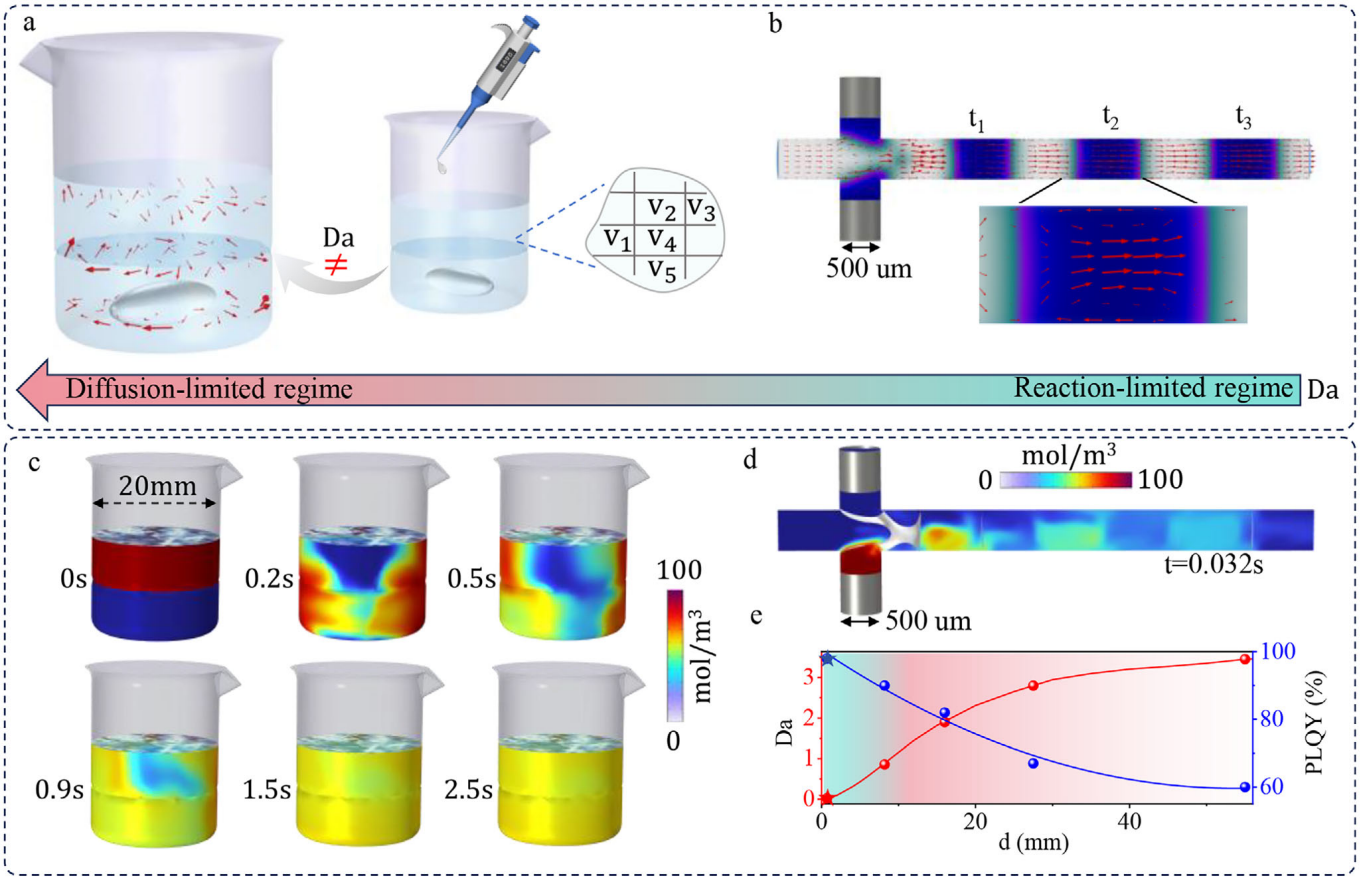
*Hydrodynamic features of the batch synthesis of PNCs via flasks and µ‐CDFS*. The calculated flow patterns and speed fields (labeled by red arrows) of batch synthesis via flasks (a) and µ‐CDFS (b). The precursors via µ‐CDFS are mixed via microscale convection in each droplet. Concentration distribution during the transport of diluted species in the flask (c) and µ‐CDFS (d). (e) Experimental PLQY of the resulting PNCs as a function of the Da number for reactors with different hydraulic diameters. The dot and star indicate the experimental PLQY are obtained from the flask reactors and µ‐CDFS, respectively.

To distinguish the inherent features between the flask‐based synthesis and µ‐CDFS, computational fluid dynamics are performed in Figure 1. When designing a system for the large‐scale synthesis of PNCs, one of the most critical parameters that needs to be considered is the Damköhler number (Da), which is defined as the dimensionless ratio of the reaction rate to the diffusion rate of precursors [7, 17, 24]. Da<1 is the desired value to conduct PNC synthesis in the reaction‐limited regime for accurate mechanistic and rate‐determining studies. In contrast, Da >1 indicates that the crystal growth is operated in an unfavorable diffusion‐limited regime. In this regime, the mixing rate is not high enough to keep up with the fast crystallization kinetics, resulting in an inhomogeneous synthesis environment and inhomogeneity in the resulting PNCs.

For large‐scale manufacturing of PNCs via the flask batch method, a large product volume is achieved by improving the reaction units in each batch. As illustrated by the speed field of batch synthesis via flasks in Figure 1a and mass transfer in Figure 1c, the enlarged reaction volume requires a longer mixing via turbulence under the high‐speed stirring. Typically, for batch synthesis with a hydraulic diameter of 20 mm, the resultant Da is >1 due to the long mixing time (>2.5 s) of precursors, leading to the reaction operating in the unfavorable flow regime. Furthermore, the scale‐up of PNCs can also be realized via accumulating reaction volume over time using µ‐CDFS. As the droplet evolution depicted in Figure 1b, one continuous phase (HF‐200 or Ar) and two dispersed phases (reagents) first meet at the cross‐junction with the flow rate of 0.02 and 0.01 mL s^−1^, and then the droplet formation occurs via the symmetrical shearing. Each droplet acts as an individual microreactor, isolated from the surrounding phase in which mixing or reaction can occur [25, 26]. The mixing time could be shortened in a second (0.032 s) via internal circulatory motions in the microchannels (Figure 1d), leading to a small Da (Da<<1). Alternatively, it means that precursors are mixed at a rate faster than they are being consumed, corresponding to a homogeneous reaction environment and thereby less heterogeneity in the resulting PNCs. As shown in Figure 1e, decreasing the hydraulic diameter (d) of flask reactors from 55 to 8 mm gradually reduces the Da values from 3.5 to 0.9, which corresponds to an enhancement in mixing efficiency. Accordingly, the PLQY of PNCs increases from 60% to ∼90%, indicating that smaller reaction volumes are more favorable for producing efficient PNCs. When the reaction volume is further reduced, operation in flask reactors becomes infeasible. Therefore, the µ‐CDFS is subsequently employed at d = 500 µm, achieving a superior PLQY of up to ∼97%.

### 2.2 Stabilizing the PNCs for Long Residence Time via Zwitterionic Ligands

**FIGURE 2:**
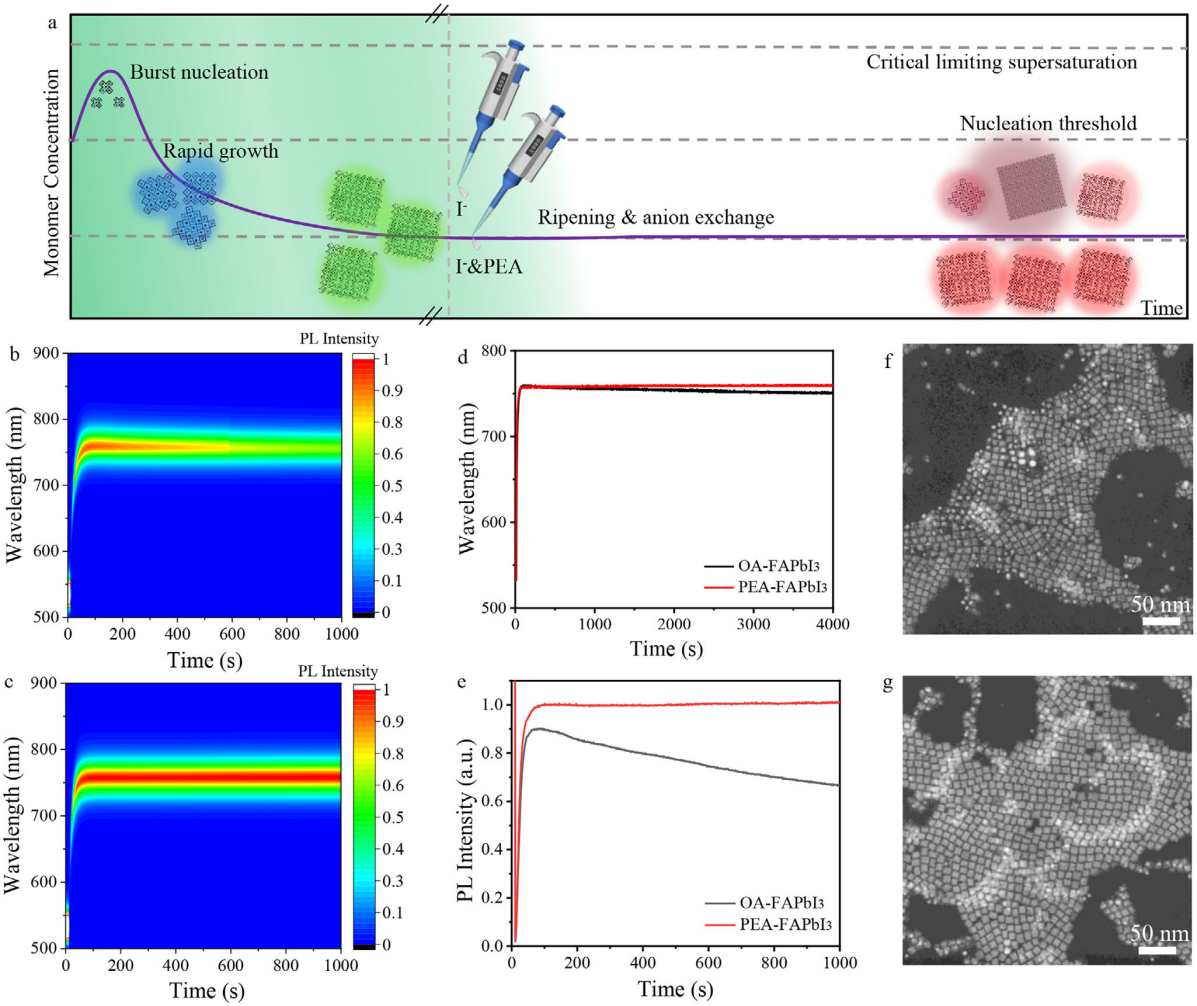
*Anti‐ripening of PNCs via zwitterionic ligands*. (a) The nucleation and growth dynamics of green‐emitting PNCs and the subsequent anion exchange reaction for color transformations: a LaMer‐inspired model. Temporal evolution of PL spectra (b,c), peak wavelength (d), and normalized PL intensity (e) of PNCs in crude solution via I‐Br anion exchange from the OA‐FAPbBr_3_ PNCs with/without simultaneous PEA ligand exchange. Typical HAADT‐STEM images of resultant FAPbI_3_ PNCs capped with the OA/OAm (f) and PEA ligands (g), respectively.

The crystallization kinetics of PNCs are described by the LaMer‐inspired model in Figure 2a. A basic prerequisite for the large‐scale preparation of PNCs via µ‐CDFS is the long residence time of as‐prepared PNCs in crude solutions. However, the PNCs are too fragile to maintain their crystal structure and optical properties due to their ionic nature. With further prolonged residence time, the decreasing supersaturation gradient will activate Ostwald ripening, thereby leading to crystal coarsening [27, 28, 29, 30]. For traditional flask‐based batch methods, the unfavorable ripening can be terminated by purifying the PNCs from the crude solution. Unfortunately, the purification is not suitable for each droplet in microfluidics due to its continuity. To improve the purity of PNCs‐based on µ‐CDFS, the Ostwald ripening of PNCs after growth should be suppressed. This can be accomplished through the following two strategies: precipitation by mixing with anti‐solvents (e.g., acetone or ethyl acetate) at the outlet, or rational ligand design to stabilize the PNCs within the crude solution itself.

The formation kinetics of PNCs can be manipulated via tailoring surface ligands. The typical long‐chain alkyl ligands (OAm, oleylamine; OA, oleic acid) undergo a reversible exothermic chemical reaction in the crude solution. The protonation of OAm leads to the formation of oleylammonium ions, which can compete with A‐site ions at the APbX_3_ NC surface and then stabilize nanocrystal growth. In addition, the presence of carboxylates in OA is crucial for the solubility of lead halides in the solvent and the reverse dissolution behavior of lead halides from PNCs. The moderate coordination of OAm and OA is suitable for controlling the nucleation and growth. However, the ligands of OAm and OA are then dynamically desorption from the surface of PNCs, subsequently resulting in the Ostwald ripening when the concentration of monomer is low. Therefore, for the synthesis of PNCs via µ‐CDFS, the ligands used to control the PNC's growth should be comprehensively redesigned. Inspired by the zwitterionic ligands with strong coordination developed by the Kovalenko group, we introduce them to stabilize the PNCs in the crude solution for scale‐up of these materials. As seen from the temporal evolution of PL spectra, peak wavelength, and normalized PL intensity of PNCs in Figure 2b–e, the FAPbI_3_ PNCs using the OA&OAm ligands are unstable after Br‐I anion exchange from the FAPbBr_3_, indicated by decreased PL intensity and blueshift of wavelength. Unlike the monodentate binding of OA and OAm, PEA ligands adopt a cross‐locked bidentate binding mode on PNC surfaces. This configuration not only inhibits the reverse dissolution of surface ions but also provides steric repulsion that minimizes solute deposition. After introducing the zwitterionic ligands of PEA in the crude PNC solution, the partial OA and OAm ligands are exchanged by PEA, as indicated by the appearance of the absorption peak at 1150–1300 cm^−1^ assigned to the PO_2_ symmetric and asymmetric stretch coordination (FTIR in Figure S1). As a result, the PL intensity and peak wavelength of PNCs are well maintained for a long residence time, demonstrating effective suppression of Ostwald ripening. Both the OA‐FAPbI_3_ PNCs and PEA‐FAPbI_3_ PNCs exhibit a similar cubic crystal structure (Figure S2). Meanwhile, the typical HAADT‐STEM images of resultant FAPbI_3_ PNCs are also characterized in Figure 2f,g. The PEA‐FAPbI_3_ PNCs show size uniformity of 8.5±2.5 nm (relative standard deviation of size distribution σ_r_ ∼16%), whereas the OA‐FAPbI_3_ PNCs exhibit a size of 8.5±3.5 nm with a large σ_r_ ∼20%.

**FIGURE 3:**
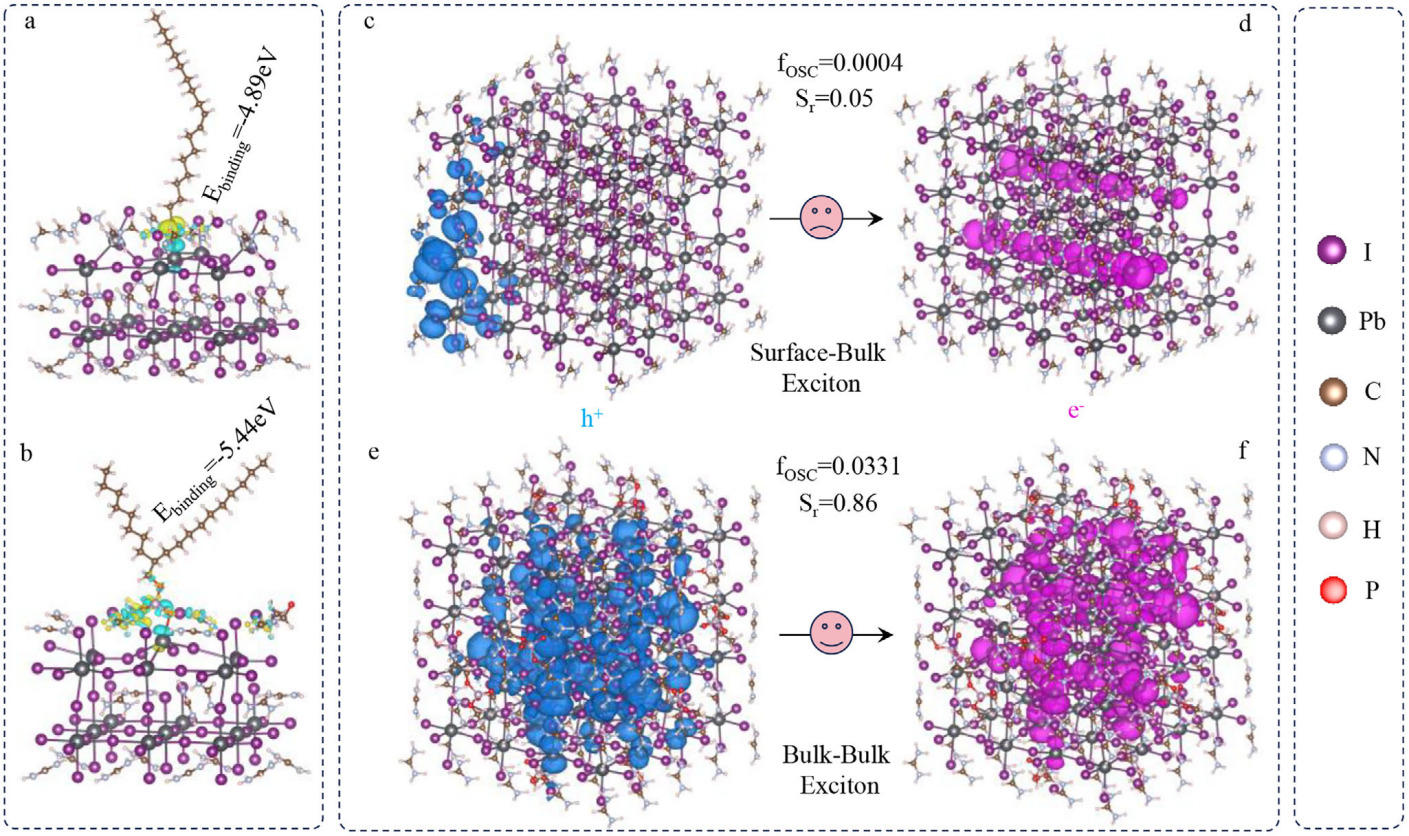
*Surface chemistry and exciton properties of PNCs capping different ligands*. Charge density difference and binding energy of the FAPbI_3_ perovskite surface capped with OA (a) and PEA (b), where yellow and blue represent the electron accumulation and depletion, respectively. The hole and electron densities of the lowest‐energy exciton in I‐vacancy (c,d) and PEA‐capped FAPbI_3_ PNCs (e,f) are calculated by TD‐DFT. The bulk‐to‐bulk exciton states are typically “bright” states with high hole–electron overlap (S_r_) and strong oscillator strength (f_osc_), and vice versa for the surface‐to‐bulk state.

To gain a further understanding of ligand binding, we modeled the interaction of surface ligands (e.g., OA and PEA) with the FAPbI_3_ surface using density functional theory (DFT). We consider a FAI‐terminated surface as representative of the perovskite surface [31]. The differential charge density of the optimized surface structure is shown in Figure 3a,b, where yellow and blue indicate regions of electron accumulation and depletion, respectively. The PEA is well designed to bind through the phosphate‐group coordination to lead atoms, along with the ammonium‐group insertion on the surface FA‐site of PNCs. The coordination is formed between the carboxylates of OA and undercoordinated Pb^2+^, as indicated by the interfacial charge transfer. The binding energy is defined as E_b_ = E_ligand+surface_– E_ligand_–E_surface_, where the latter three terms are the energy of the surface and bound ligand, the energy of the isolated ligand, and the energy of the bare surface, respectively. Our calculated binding energy for PEA, at −5.44 eV, is large compared to the calculated values for OA (−4.89 eV). The large binding energy is a result of contributions from anion and cation binding moieties, enabling the cross‐locking of the perovskite surface. To characterize the nature of the exciton states, we further calculated the hole and electron densities of the lowest‐energy exciton in I‐vacancy and PEA‐passivated FAPbI_3_ PNCs by the TDDFT in Figure 3c–f. For the I‐vacancy FAPbI_3_ PNCs, the distribution of hole and electron density is asymmetric, where the holes are mainly located at the surface and the electrons at the corner, exhibiting a feature of surface‐to‐bulk exciton. On the other hand, the hole and electron density for the PEA‐passivated FAPbI_3_ PNCs are uniformly distributed in the bulk, showing a bulk‐to‐bulk exciton. The hole and electron overlap are estimated by S_r_ [32], which is defined as

$$S_{r}\;\left(r\right)=\sqrt{\rho^{\mathrm{hole}}\left(r\right)\rho^{\mathrm{ele}}\left(r\right)}$$

where ρ^hole^ and ρ^ele^ represent the hole and electron density, respectively. In comparison with the surface‐to‐bulk states in I‐vacancy FAPbI_3_ PNCs, the S_r_ for bulk‐to‐bulk states in PEA‐passivated FAPbI_3_ PNCs have a great increase from 0.05 to 0.86, indicating the high overlapping between electron and hole wave functions. Meanwhile, the PEA‐passivated FAPbI_3_ PNCs have a higher oscillator strength (0.0331) than I‐vacancy FAPbI_3_ PNCs (0.0004). Those bulk‐to‐bulk states with high oscillator strength are typically “bright” states, indicating a potential high PLQY value [33, 34, 35, 36, 37].

The ultrafast femtosecond transient absorption (TA) measurements are shown in Figures S3–S5. The TA spectra of OA‐FAPbI_3_ and PEA‐FAPbI_3_ PNCs show excitonic bleach at 726 and 733 nm, respectively. The bleach curves are well fitted by bi‐exponential functions with one ultrafast component (∼ps) arising from defect‐related non‐radiative recombination and the other long component (∼ns) corresponding to the radiation recombination. The bleach recovery dynamics at the beginning are relatively slower for PEA‐FAPbI_3_ PNCs than OA‐FAPbI_3_, indicated by the ultrafast components increasing from 74 to 188 ps. The contribution of the ultrafast lifetime component is much lower (23%) in PEA‐FAPbI_3_ PNCs vs. 41% in the pristine system. It is thus evident that PEA ligands play an important role in eliminating the surface defects via reducing the octahedral distortion [38]. Meanwhile, the long component of PEA‐FAPbI_3_ PNCs is shorter than OA‐FAPbI_3_ PNCs (4.69 vs. 5.89 ns), indicating a high exciton energy [39]. Moreover, the time‐resolved PL spectra of OA‐FAPbI_3_ and FEA‐FAPbI_3_ PNCs is measured in Figure S6. The high contribution of a short‐lived lifetime leads to the OA‐FAPbI_3_ PNCs with a faster average lifetime of 3.17 ns. The FEA‐FAPbI_3_ PNCs show single exponential properties with a longer lifetime of 19.4 ns. An increase in average PL lifetime is accompanied by enhanced PLQY, indicating passivation of nonradiative trap centers to a great extent.

### 2.3 Synthesis of Visible PNCs via Continuous Droplet Microfluidics

**FIGURE 4:**
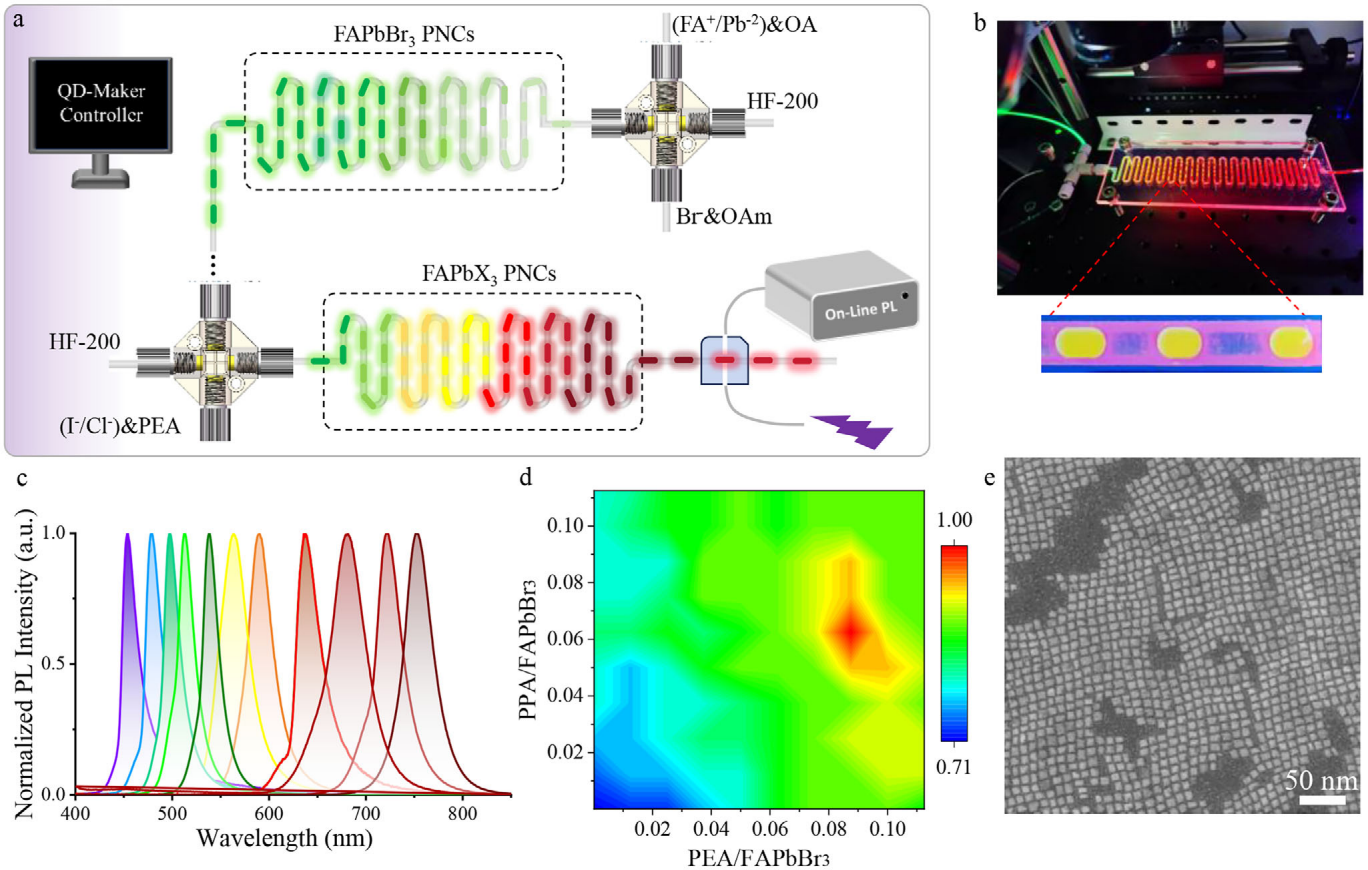
*Synthesis of PNCs via continuous droplet microfluidics*. (a) Illustration of the µ‐CDFS platform integrated with online photoluminescence detection for the synthesis and real‐time characterization of PNCs. (b) Image of the generated droplets under UV excitation (λ_ex_ = 365 nm), showing bright PL of PNCs. (c) Online fluorescence spectra of FAPbX_3_ PNCs spanning the whole visible spectral region with narrow emission. (d) High‐throughput screening of PNCs by varying the molar ratios of the PPA and PEA ligands. (e) HAADT‐STEM images of FAPbI_3_ PNCs synthesized via µ‐CDFS.

The setup of µ‐CDFS in this work is designed with two units (Figure 4a; Figure S7): (i) the former is to obtain the green‐emitting FAPbBr_3_ PNCs via controlling the nucleation and growth, and (ii) the latter is to perform the color translation on demand via anion exchange. The PEA ligands are introduced into the crude solvents at the second unit. Experimentally, the precursor of FA^+^ and Pb^2+^ dissolved in n‐octane using OA as ligands is quickly mixed with the precursor of OAM‐I in n‐octane via a cross‐junction, and then the droplets are generated via the shear force of the carrier fluid of HF‐200 or Ar. This setup provides 0.8 g h^−1^ per channel production, exhibiting great potential for scale‐up of these materials. The digital photograph of the generated droplets with bright PL under UV excitation (λ_ex_ = 365 nm) is shown in Figure 4b. The µ‐CDFS system exhibits excellent repeatability and consistency, as demonstrated by the in situ PL mapping recorded at the outlet in Figure S8. To tune the color, the as‐prepared FAPbBr_3_ PNCs are subsequently injected into the second cross‐junction with mixed halide precursors. As illustrated in Figure 4c and Figure S9, the color of PNCs can cover the visible region (453–753 nm) with narrow emission. Moreover, to improve adaptability between the perovskite surface and PEA ligand during the anion exchange, the head‐group affinity should be comprehensively considered. For instance, when performing I‐Br anion exchange from FAPbBr_3_ PNCs, adjusting the head group of zwitterionic ligands aids in matching the larger lattice constant of a series of resultant iodine‐rich PNCs. The spacing of head group between the ammonium and phosphate groups can be tuned via the length of the carbon bridge: PEA has a two‐carbon bridge, while PPA has a three‐carbon bridge. For parent FAPbBr_3_ PNCs, the anionic and cationic moieties of PEA is well geometric fitness into the surface lattice sites of PNCs. With the progression of I‐Br exchange reaction, the lattice constant gradually expands, giving rise to a decrease in fitness. The co‐addition of PPA and PEA enables synergistic coordination with the surface ions of PNCs, thereby maintaining their emission and stability. The high‐throughput screening of PPA and PEA is performed via an automated syringe pump‐based flow control and in situ PL characterization. As shown in Figure 4d, the optimized PEA/PPA molar ratio of 1.45. The HAADT‐STEM images of FAPbI_3_ PNCs show a high size uniformity of 8.5±2 nm with σ_r_ <14%, indicating an excellent monodispersity (Figure 4e; Figures S10 and S11). Furthermore, the µ‐CDFS is a general platform for PNC synthesis, which can also be utilized to prepare all‐inorganic CsPbX_3_ PNCs with the emission covering the visible region (Figures S12–S14).

### 2.4 Applications of High‐Gamut PNCs for Backlit Displays

**FIGURE 5:**
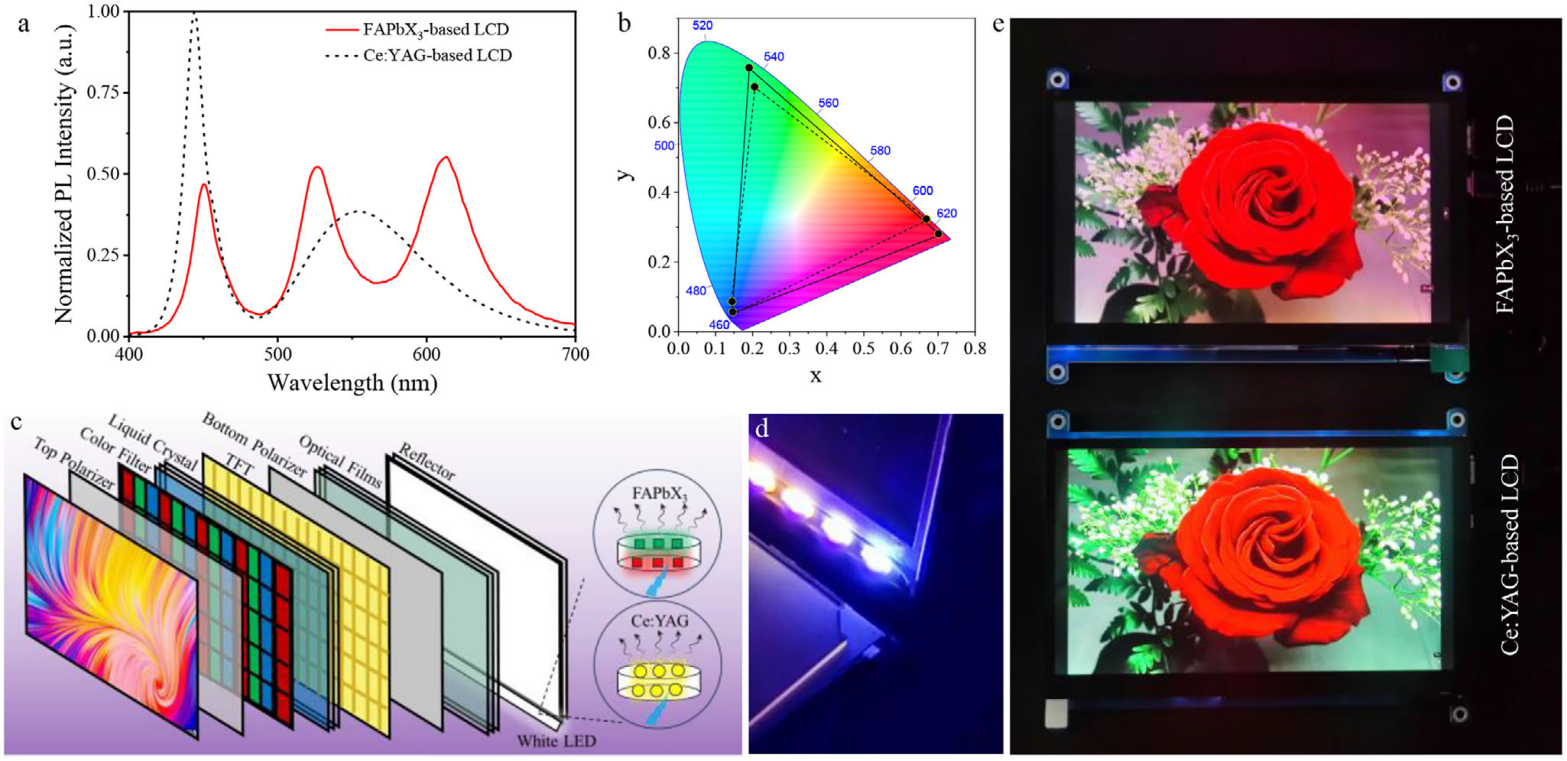
*Applications in backlit displays of PNCs synthesized via µ‐CDFS*. (a) white‐light PL spectra of the backlight based on FAPbX_3_ PNCs or Ce: YAG phosphor. (b) CIE color coordinates of the backlight in Figure 5a (solid black triangle: our work, dashed triangle: NTSC standard). (c) LCD structure of the backlight module based on PNCs or Ce: YAG. (d) A photograph of a white‐light backlight using PNCs as color conversion layers. (e) a 7‐inch display manifested by our and commercial backlights.

The PNCs have been demonstrated as potential candidates for color conversion in backlit displays. As shown in Figure 5a, the assembly backlight unit yields bright white light using the blue LED to excite green and red‐emitting PNCs. The corresponding EL spectra exhibit 450 nm (blue LED chips), 526 nm (green FAPbBr_3_ PNCs), and 614 nm (red FA_0.2_Cs_0.8_Pb(Br/I)_3_ PNCs) tricolor narrowband emissions. To avoid detrimental color segregation via anion exchange, the green and red PNCs are separately dispersed in PETMP/TAIC monomers and photocured layer‐by‐layer according to our previous works [40]. As a comparison, the commercial backlight is generally combined with the blue chips and yellow Ce: YAG phosphor with a broadband emission. The color gamut of our backlights was compared with that of commercial backlights and the standard of NTSC. The RGB color coordinates for this work and the NTSC are (0.71, 0.28), (0.16, 0.78), (0.15, 0.05), and (0.67, 0.33), (0.21, 0.71), (0.14, 0.08), respectively. The area of the RGB triangle of our white light covered 126% of the NTSC color space (Figure 5b). Furthermore, the stability of backlight is recorded at different luminance (Figure S15). The backlight exhibits negligible degradation at 10^4^ nits over 60 min, meeting the threshold values of lighting and displays [41]. However, a slight degradation is initially observed at the luminance reached at 10^5^ nits, which becomes significant as luminance further increases to 10^6^ nits. This degradation under intense illumination is attributed to the thermal activation of defects, phase segregation, or agglomeration of PNCs [42]. A wide color gamut display prototype (Figure 5c) is successfully integrated with a commercial TFT‐LCD panel and the present PNCs‐based white‐lighting backlight (Figure 5d). Compared with the commercial LCD using Ce: YAG phosphor, the LCD screens adopting PNC‐based backlight exhibit more details of object colors. For the display of a flower, the commercial LCD screen presents only the deep‐red, while our assembly backlight unit screen demonstrates a more remarkable color rendition (pure deep‐red color and pink color). Meanwhile, a pure green color and a high saturation are realized for our PNC‐based display (Figure 5e).

## 3 Conclusion

In conclusion, we have developed a general strategy for large‐scale preparation of PNCs through the integration of a microfluidic droplet flow synthesis (µ‐CDFS) with zwitterionic molecular engineering. The introduction of zwitterionic PEA&PPA ligands effectively suppressed Ostwald ripening, enabling extended stability of the fresh PNCs in crude solutions. Meanwhile, the enhanced microscale convection within each droplet significantly improved the mixing efficiency of precursors. Based on the above, the µ‐CDFS achieved a large‐scale synthesis rate of 0.8 g h^−1^ per channel while maintaining uniform size distribution (σ_r_ <14%) and high PLQY >97%. The emission wavelength of PNCs could be tuned on demand in the visible region (453–753 nm) via halide anion exchange. The LCD with a wide color gamut to 126% of the NTSC was fabricated by adopting R&G PNC‐based backlight. This work establishes an autonomous platform for large‐scale production of high‐quality PNCs and further advances their commercial applications in display technologies.

## Conflicts of Interest

The authors declare no conflicts of interest.

## Supporting information




**Supporting File**: advs74096‐sup‐0001‐SuppMat.docx.

## Data Availability

The data that support the findings of this study are available from the corresponding author upon reasonable request.
