# Supplementary material for: Large‐Scale Synthesis of Monodispersed Perovskite Nanocrystals via Autonomous Continuous Droplet Microfluidics
Source: Adv Sci (Weinh). 2026 Jan 31;13(19):e24155. doi: 10.1002/advs.202524155 (PMC13045447; doi:10.1002/advs.202524155)
Supplement: Supplementary file 1 — Supporting File: advs74096‐sup‐0001‐SuppMat.docx. [file ADVS-13-e24155-s001.docx]

Electronic Supporting Information

Large-Scale Synthesis of Monodispersed Perovskite Nanocrystals via Autonomous Continuous Droplet Microfluidics

*Guangguang Huang, Xiangyu Liu, Long Song, Hailong Feng, Zuliang Du^*^*

National & Local Joint Engineering Research Center for High-efficiency Display and Lighting Technology, Key Laboratory for Special Functional Materials of Ministry of Education, School of Nanoscience and Materials Engineering, Henan University, Kaifeng, China

E-mail: zld@henu.edu.cn

Materials and Methods

*Chemicals*: PbBr_2_ (99.999%), Cs_2_CO_3_ (99.9%), octanoic acid (99%), zinc chloride (ZnCl_2_, 99.99%), tetrabutylammonium bromide (TOAB, 98%), ethylacetate (99.8%), zinc iodine (ZnI_2_, 99.99%), n-octane (99%), and didodecyldimethylammonium bromide (DDAB, 98%) were purchased from Macklin. Oleylamine (OAm, technical grade, 70%), oleic acid (OA, technical grade, 90%), formamidine acetate salt (FA-AC, 99%), lead(II) acetate trihydrate (99.999%), and bromotrimethylsilane (97%) were purchased from Sigma Aldrich. Triethylamine (99.5%), tetrahydrofuran (THF, 99.5%), 2-octyl-1-dodecanol (97%), 2-aminoethan-1-ol (99.5%), acetic acid (99.99%), and 3-aminopropan-1-ol (99%) were purchased from Aladdin. All the chemicals were used directly as received.

*Synthesis of zwitterionic PEA and PPA*: The PEA synthesis route starting with alcoholysis of phosphorous oxychloride was adopted from the previously reported work.[^1^](#_ENREF_1) Solution of 2-octyl-1-dodecanol (0.025 mol) dissolved in THF (25 ml), along with triethylamine (0.275 mol), was added dropwise under vigorous stirring into a solution of phosphorous oxychloride (0.03 mol) in THF (2.5 ml) on an ice-water bath. The reaction mixture solution was subsequently kept at 20 °C for 15 min to complete the reaction. Next, 2-aminoethan-1-ol (0.03 mol) and triethylamine (0.06 mol) in THF (37.5 ml) were added to the reaction mixture kept in a room-temperature water bath. Subsequently, the mixture was heated to 40 °C for 20 min to complete the ring closure. Finally, the reaction mixture was filtered and dried to remove precipitated triethylamine hydrochloride. An oily residue, alkyl-2-oxo-1,2,3-oxazaphospholane, was then dissolved in a mixture of acetic acid (5.7 ml) and distilled water (2.6 ml) at 70 °C. After 40 min, ring scission at the P-N bond was complete, and the product was finally separated by beating with acetone (~140 ml). 1-octyl-2-dodecanol-3-phosphoethanolamine (PEA) was collected and dried overnight under a vacuum at 45 °C. For PPA, 3-aminopropan-1-ol was used instead of 2-aminoethan-1-ol.

*Flask-based synthesis of FAPbX_3_ nanocrystals*: A precursor was prepared by loading 0.2 mmol lead(II) acetate trihydrate, 0.75 mmol FA-AC, and 2 mL dried-OA into 4 mL octane, and the mixture was stirred for 30 min at 60 ^o^C to form a clear solution. The B precursor solution was prepared by dissolving 0.4 mmol bromotrimethylsilane (TMSBr) and 0.4mmol OAm into 6mL of octane. The FAPbBr_3_ nanocrystals were formed via quickly injecting the B precursor into the A precursor solution under vigorous stirring. All the processes were performed in a 20 ml flask at room temperature. The halide anion-exchange solution was prepared by mixing 100 mg ZnI_2_ and 200 μL OAm in 10 mL of octane, and then, the mixture was stirred for 24 h. For the FAPb(Br/I)_3_ nanocrystals, an appropriate amount of ZnI_2_-OAm anion-exchange solution was further injected into the above FAPbBr_3_ solution. For the FAPb(Br/Cl)_3_ nanocrystals, the ZnI_2_ in the anion-exchange solution was replaced by equal molar ZnCl_2_.

*Synthesis of the FAPbX_3_ nanocrystals via* *μ-CDFS*: A precursor was prepared by loading 0.2 mmol lead(II) acetate trihydrate, 0.75 mmol FA-AC, and 2mL dried-OA into 4 mL octane, and the mixture was stirred for 30 min at 60 ^o^C to form a clear solution. The B precursor solution was prepared by dissolving 0.4 mmol TMSBr and 0.4mmol OAm into 6mL of octane. The anion-exchange solution was prepared by mixing 100 mg ZnI_2_ and 200 μL OAm in 10 mL of octane, and then, the mixture was stirred for 24 h. The preparation of PNCs was carried out at room temperature via the μ-CDFS platform shown in Figure 4a. Fluorinated ethylene propylene (FEP) tubing (500 μm ID, 1.6 mm OD) was utilized to connect the above precursor syringes to a polyether ether ketone (PEEK) four-way cross-junction. The A and B precursors were injected into the cross-junction at a speed of 0.01 mL/s. The HF-200 carrier was injected at a speed of 0.02 mL/s. The A and B were then mixed via the microscale convection in each droplet. For the FAPb(Br/Cl)_3_ and FAPb(Br/I)_3_ PNCs, the formed FAPbBr_3_ parent solution was injected into the second cross-junction, and then mixed with an anion-exchange precursor at a certain rate. To suppress the Ostwald ripening, the zwitterionic ligands PEA and PPA were introduced into the second cross-junction.

*Synthesis of the CsPbX_3_ nanocrystals via* *μ-CDFS*: The synthesis of CsPbX_3_ nanocrystals was according to our previously reported work.[^2^](#_ENREF_2) Cesium precursor was prepared by loading 0.5 mmol of Cs_2_CO_3_ and 2 mL of OTA into an 18 mL toluene, and the mixture was stirred for 10 min at 60 ^o^C. The PbBr_2_ precursor solution was prepared by dissolving 1 mmol of PbBr_2_ and 2 mmol of TOAB in 20 mL of toluene. The cesium precursor solution (0.01 mL/s) was swiftly mixed with the PbBr_2_ precursor (0.02 mL/s) via the cross-junction. The carrier HF-200 was injected into the cross-junction at a speed of 0.03 mL/s. Subsequently, the emission wavelength was tuned by injecting the anion-exchange solution into the second cross-junction at a certain rate along with the PEA solution.

*Fabrication of backlit displays*: The color-conversion ink was prepared by dispersing the green- and red-emitting PNCs in a PETMP-TAIC photoresist according to our previous work.[^3^](#_ENREF_3) Then, color-conversion ink was dropped on the top of the blue LED chips and photocured in situ. The 7-inch LCD was realized via the above integrated white backlights and a thin-film transistor driver (Raspbian) together.

*DFT calculations*: Density functional theory (DFT) calculations for surface slab models, including binding energy and charge density difference (CDD), were done with the open CP2K computational package utilizing a dual basis of localized Gaussians and plane waves (GPW). Goedecker-Teter-Hutter (GTH) pseudopotentials in the generalized gradient approximation (GGA) with the Perdew-Burke-Ernzerhof (PBE) exchange-correlation functional were used. A grid charge density cut-off of 500 Ry was used. The vacuum thickness between the perovskite layers was set to 35 Å. The Kohn-Sham orbitals were described in the DZVP-MOLOPT-SR-GTH basis set, and the electron density was expanded in a plane wave basis set with a kinetic energy cutoff of 60 Ry. The PNC models were cut from the bulk FAPbI_3_ with nearly bulk shape (i.e., characterized by side length L). Simulations were performed with periodic boundary conditions in a 50.3 Å^3^ unit cell for a L=30.5 Å PNC. The excitonic properties were further studied by the TD-DFT combined with Grimme’s D3 (BJ) dispersion correction. The electron excitation analysis, electron and hole densities of the lowest exciton, along with the f_osc_ and S_r_, were generated using the multifunctional wavefunction analyzer (Multiwfn).

*Flow dynamics simulations*: for flask-based batch synthesis of PNCs, the two-phase flow with level-set for the CFD module was used. The simulations were carried out in a two-dimensional domain, as shown in **Figure 1**. The first phase at the top of the flask was air, and the second phase at the bottom was octane (incompressible Newtonian fluids). The walls were specified as a wetted wall condition with a contact angle (45 degrees) for all the cases. The stirring speed was set to 360 rpm. For the droplet-based in-flow synthesis, a + junction pipe micromixer with an inner diameter of 500 μm was selected to investigate the effect of the slug and the liquid film. The boundary conditions at the outlet and wall were set as the same in all simulations. The outlet was set as a pressure boundary with a gauge pressure of 0 Pa. A no-slip condition was applied at the walls, ensuring that the velocity is zero. The first phase was octane as the reaction medium with an injection speed of 0.01 ml/s for the top/down inlets. The second phase was HF-200 as a carrier with an injection speed of 0.02 ml/s for the left inlet. After the two phases intersect, a liquid droplet flow would form. The speed fields for the batch and in-flow synthesis were labeled with red arrows. The flow is laminar and governed by the Navier-Stokes equations. A transient solver was employed along with the volume of fluid method for modeling the multiphase system. The mass transfer was simulated using the transport of diluted species (TDS). The mass flux was further given by diffusion and convection via coupling the speed field, which was solved from the Navier-Stokes equations.

**Figure S1**. FT-IR spectra of OA-FAPbI_3_ PNCs and PEA-FAPbI_3_ PNCs.

**Figure S2**: Powder XRD patterns of OA-FAPbI_3_ PNCs and PEA-FAPbI_3_ PNCs.


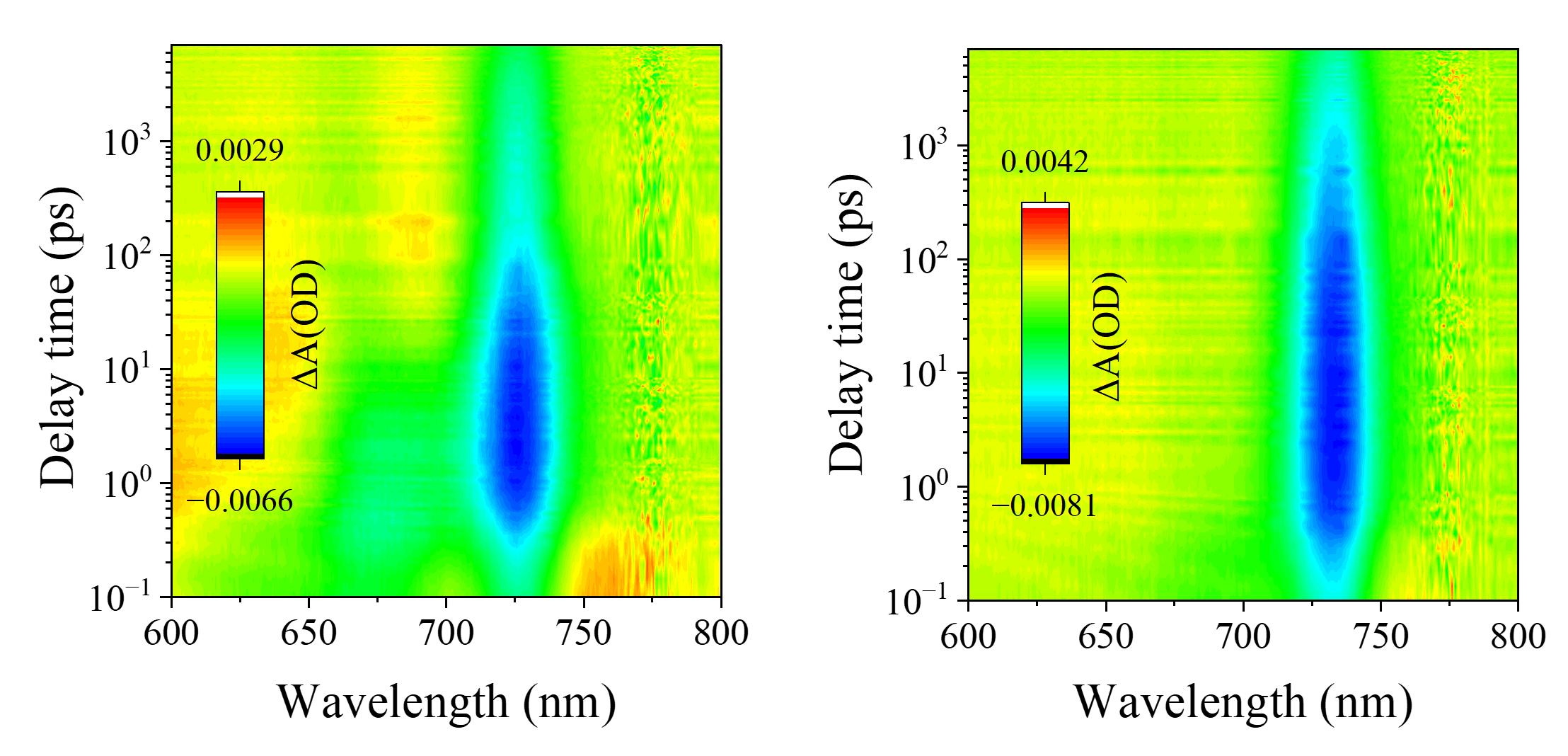


**Figure S3**. Normalized transient absorption (ΔA) spectra of OA-FAPbI_3_ PNCs (a) and PEA-FAPbI_3_ PNCs (b) under pump fluence of 1.5 μW (375 nm).

**Figure S4**. Time evolution of the ΔA measured at the emission peak of OA-FAPbI_3_ PNCs and PEA-FAPbI_3_ PNCs.

**Figure S5**. Photophysical diagrams describing the carrier recombination in OA-FAPbI_3_ PNCs after the PEA to OAm&OA ligand exchange.

**Figure S6**. Time-resolved PL spectra and their fitting results of OA-FAPbI_3_ and FEA-FAPbI_3_ PNCs, respectively.


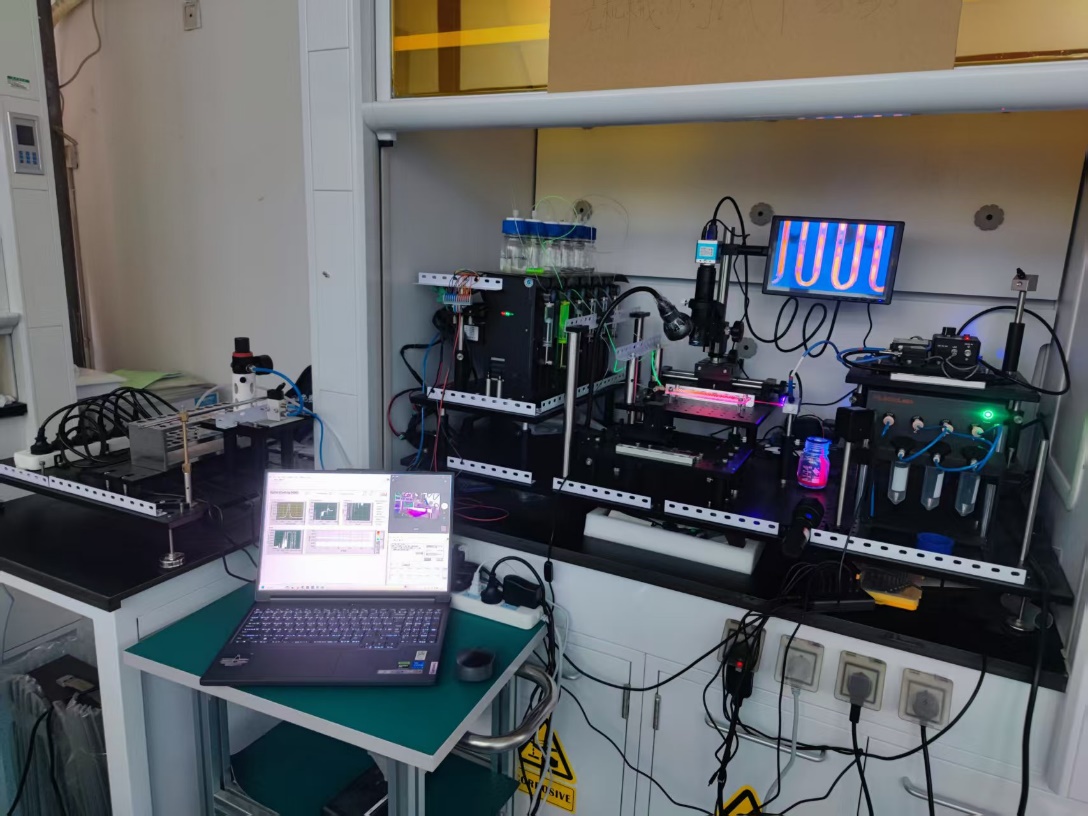


**Figure S7**. Digital photograph of the microscale continuous droplet-in-flow synthesis (*μ-CDFS*) for color translation of PNCs via anion exchange.

**Figure S8**: Consistency evaluation of PNC synthesis via μ-CDFS.

**Figure S9**. Digital photograph of colloidal FAPbX_3_ PNCs in n-octane.


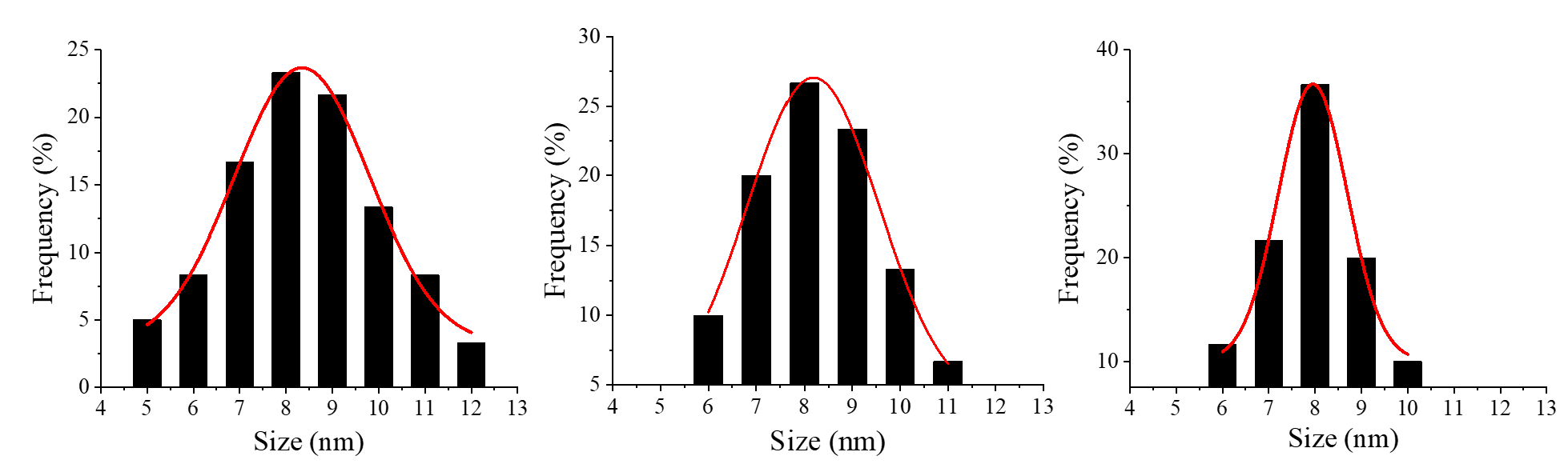


**Figure S10**. Statistical histogram of the size distribution of PNCs capped with different ligands: left (OAm&OA), median (PEA), and right (PEA&PPA).

**Figure S11**. Relative standard deviation of size distribution σ_r_ of PNCs capped with different ligands.

**Figure S12**: Normalized PL spectra of colloidal full-color CsPbX_3_ PNCs in n-octane.

**Figure S13**: Digital photograph of colloidal CsPbX_3_ PNCs in n-octane.

**Figure S14**: Normalized PL intensity of OA&OAm-CsPbI_3_ and PEA&PPA-CsPbI_3_ PNCs.

**Figure S15**: Stability evaluation of the white-light backlight operated at different luminance.

1. V. Morad, A. Stelmakh, M. Svyrydenko, L. G. Feld, S. C. Boehme, M. Aebli, J. Affolter, C. J. Kaul, N. J. Schrenker, S. Bals, Y. Sahin, D. N. Dirin, I. Cherniukh, G. Raino, A. Baumketner and M. V. Kovalenko, *Nature*, 2024, **626**, 542-548.

2. G. Huang, K. Sun, X. Xiong, L. Song, G. Liu, C. Zheng, F. Yu, and Z. Du, *Laser Photonics Rev.*, 2024, **19**, 2401121_._

3. G. Huang, F. Zhang, X. Xiong, K. Sun, H. Ruan, C. Wang, C. Li, Y. Zhao, M. Li, G. Cheng, and Z. Du, *Adv Mater*, 2025, **37**, e2411453.
